# Gelatin-Coated High-Sensitivity Microwave Sensor for Humidity-Sensing Applications

**DOI:** 10.3390/s24196286

**Published:** 2024-09-28

**Authors:** Junho Yeo, Younghwan Kwon

**Affiliations:** 1 Department of Artificial Intelligence, Daegu University, 201 Daegudae-ro, Gyeongsan-si 38453, Republic of Korea; 2 Department of Energy System Engineering, Daegu University, 201 Daegudae-ro, Gyeongsan-si 38453, Republic of Korea; y_kwon@daegu.ac.kr

**Keywords:** gelatin, polyvinyl alcohol, defected ground structure with a modified interdigital capacitor (DGS-MIDC), microwave humidity sensor, high sensitivity

## Abstract

In this paper, the humidity-sensing characteristics of gelatin were compared with those of poly(vinyl alcohol) (PVA) at L-band (1 ~ 2 GHz) microwave frequencies. A capacitive microwave sensor based on a defected ground structure with a modified interdigital capacitor (DGS-MIDC) in a microstrip transmission line operating at 1.5 GHz without any coating was used. Gelatin is a natural polymer based on protein sourced from animal collagen, whereas PVA is a high-sensitivity hydrophilic polymer that is widely used for humidity sensors and has a good film-forming property. Two DGS-MIDC-based microwave sensors coated with type A gelatin and PVA, respectively, with a thickness of 0.02 mm were fabricated. The percent relative frequency shift (PRFS) and percent relative magnitude shift (PRMS) based on the changes in the resonant frequency and magnitude level of the transmission coefficient for the microwave sensor were used to compare the humidity-sensing characteristics. The relative humidity (RH) was varied from 50% to 80% with a step of 10% at a fixed temperature of around 25 °C using a low-reflective temperature and humidity chamber manufactured with Styrofoam. The experiment’s results show that the capacitive humidity sensitivity of the gelatin-coated microwave sensor in terms of the PRFS and PRMS was higher compared to that of the PVA-coated one. In particular, the sensitivity of the gelatin-coated microwave sensor at a low RH from 50% to 60% was much greater compared to that of the PVA-coated one. In addition, the relative permittivity of the fabricated microwave sensors coated with PVA and gelatin was extracted by using the measured PRFS and the equation was derived by curve-fitting the simulated results. The change in the extracted relative permittivity for the gelatin-coated microwave sensor was larger than that of the PVA-coated one for varying the RH.

## 1. Introduction

Humidity refers to the amount of water vapors existing in the air [1], and it is an important physical quantity that evaluates air quality along with temperature. An accurate measurement of humidity is necessary because human quality of life, human health, and the environment, as well as products and processes in various industrial fields are significantly affected by humidity along with temperature [2]. Two commonly used definitions of humidity are absolute humidity and relative humidity (RH). The RH has been widely used for humidity measurements because of RH sensors’ simplicity and affordability [3]. Based on the transduction techniques or sensing methods, RH sensors can be classified as resistive, capacitive, optical, gravimetric, piezoresistive, and magnetoelastic [4]. Among the various RH measurement techniques, the electrical properties of humidity-sensing materials, such as resistance (or electrical conductivity) and capacitance, were the most commonly used [5]. Resistive-type humidity sensors are based on the change in the resistance or conductivity of the sensing material for the different RHs, whereas capacitive-type humidity sensors are associated with the change in capacitance of the sensing material through the variations in relative permittivity. In addition, materials for resistive-type humidity sensors can be divided into low-resistance and high-resistance materials. Note that high-resistance materials are affected by both the resistance and capacitance changes, and that the humidity sensors that incorporate high-resistance materials are called impedance-type sensors.

The humidity-sensing characteristics of the RH sensors mainly depend on the microscopic structure of the material used as the humidity-sensing layer, such as the size of the pores, the uniformity of the surface morphology, or the thickness of the layer [1,2]. It is well known that materials with porous and irregular surface morphologies show a higher humidity-sensing ability because the porous and irregular surface areas provide more empty spaces for the absorption of the hydroxyl group and hydronium ions in water molecules. The RH-sensing materials can be classified as polymers, ceramics, carbon-based materials, and composite and hybrid materials.

Porous ceramics, such as metal oxides and spinel- and perovskite-type oxides, have been used for the humidity sensors [6]. Alumina (Al_2_O_3_)-, Zinc (Zn)-, tin (Sn)-, anatase (TiO_2_, titanium oxide)-, tungsten (W)-, hematite (Fe_2_O_3_), and cobalt (Co_3_O_4_)-based oxides are commonly used to create metal oxides [7,8,9,10,11,12,13,14]. Perovskite-type ceramics, such as BaTiO_3_, PbTiO_3_, CaTiO_3_, LaFeO_3_, ZnSnO_3_, and NaTaO_3,_ have been used in humidity sensors [15,16,17,18,19,20,21]. MgAl_2_O_4_, MgFe_2_O_4_, MgCr_2_O_4_-TiO_2_, and ZnCr_2_O_4_-k_2_CrO_4_ are spinel-type ceramics used to create humidity sensors [22,23,24,25]. Oxygen-free ceramics, such as zeolites and clay materials, were also used for the humidity sensors [26,27].

Carbon-based materials, such as carbon nanotubes (CNTs), carbon nano-coils (CNCs), and graphene oxide (GO), have been widely used in humidity sensors. CNTs are seamless cylinders consisting of one or several graphene layers and can be either single-walled (SWCNTs) or multi-walled (MWCNTs) [28]. CNTs can absorb large amounts of water molecules due to their very large surface area-to-volume ratio and their porous structures when used for humidity sensors [29]. CNCs are quasi-one-dimensional materials with unique helical morphology and can provide abundant spaces for the adsorption of water molecules for humidity sensing [30]. Graphene is a two-dimensional monolayer of sp^2^-bonded carbon atoms, whereas GO is a graphene derivative with carbon atoms linked to oxygen functional groups. The addition of oxygen functional groups results in GO having excellent absorption properties when used in humidity sensors [31].

Polymers have been most widely used for humidity-sensing materials because of their advantages, such as their low cost, easy fabrication process, and their good stability [32]. Polymer humidity sensors can also be categorized into the resistive type and the capacitive type. The resistive-type polymer humidity sensors are divided into the ionic-conduction type and the electronic-conduction type. The ionic-conduction polymers are polyelectrolytes, which are polymers with electrolytic groups, such as quaternary ammonium salt, sulfonate salt, and phosphonium salt [33,34,35]. The electronic-conduction types are conducting/semiconducting polymers such as polyaniline (PANI) [36], poly(p-diethynylbenzene) (PDEB) [37], poly(3,4-ethylenedioxythiophene) (PEDOT) [38], and poly(3,4-ethylenedioxythiophene)–poly(styrene-sulfonate) (PEDOT-PSS) [39].

Various capacitive-type polymers have been used for RH sensors due to their advantages, such as their simpler fabrication, rapid responses, small hysteresis, and their excellent stability, compared to resistive-type polymers [32]. The capacitive-type polymers are insulating polymers whose permittivity varies depending on the amount of absorbed water as a function of humidity, such as poly(methyl methacrylate) (PMMA), polyimide, poly(2-hydroxyethyl methacrylate) (PHEMA), and poly(vinyl alcohol) (PVA) [40,41,42,43]. It was reported in the literature that the humidity-sensing sensitivity of PVA is higher than polyimide, PMMA, and PHEMA [44]. Cellulose derivatives, such as cellulose acetate butyrate (CAB) or cellulose nanofibers (CNF), have also been used for capacitive humidity sensors [45].

**Figure 1:**
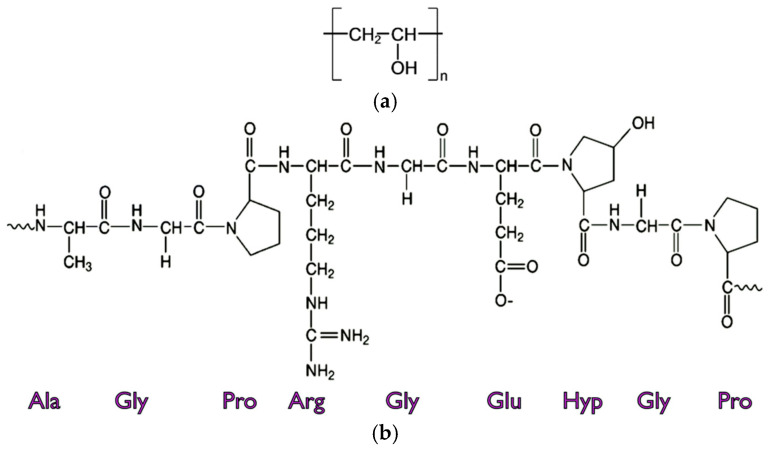
Chemical structures of (**a**) PVA and (**b**) gelatin.

Gelatin is a natural polymer based on protein sourced from collagen, which is mainly obtained from the skins, connective tissues (tendons and ligaments), and bones of cows and pigs [46]. In general, gelatin can be divided into two types by using raw materials and the pre-treatment applied during the manufacturing process: type A with an acid treatment, and type B with an alkaline treatment. Recently, gelatin’s humidity-sensing ability has been studied because of its hygroscopic characteristics with biocompatibility and biodegradability. The changes in the resistance of a gold interdigitated electrode (IDE) structure coated with gelatin polymer were measured when the RH varied from 15% to 86% [47]. The performance of gelatin as a capacitive humidity-sensing material was examined with the changes in the capacitance of a planar IDE coated with gelatin when the RH was ramped up and down between 50% and 90% with a step of 10% [48]. However, these characteristic studies were conducted in the low-frequency band, and a comparison of the humidity sensitivity for gelatin with other polymers using a capacitive microwave sensor has never previously been studied in microwave frequency bands (0.3 to 300 GHz). The chemical structures of PVA [49] and gelatin [50] are shown in Figure 1. PVA is a water-soluble synthetic polymer consisting of a carbon chain as its backbone, and a hydroxyl (OH) functional group, and has various desirable properties, such as a high hydrophilicity, biocompatibility, and good film-forming and process abilities [51]. Gelatin is a natural polymer with a heterogeneous mixture of single- or multi-stranded polypeptides containing between 300 and 4000 amino acids [52]. The amino acid compositions of gelatin are 26~30% of glycine (Gly), 14~18% of proline (Pro), 13~15% of hydroxyproline (Hyp), 11~12% of glutamic acid (Glu), 8~10% of alanine (Ala), 8~9% of arginine (Arg), and other amino acids [53]. The hydroxyl functional group in hydroxyproline might play an important role in the humidity-sensing capability along with the porous structure of gelatin.

Planar microwave technology-based sensors have been widely used because of their advantages, such as their low profile, low cost, their simple structure, and their ease of fabrication [54]. Among various methods, the resonant frequency-based methods using planar microwave resonators, such as the split-ring resonator (SRR) or the complementary SRR (CSRR), implemented on microstrip transmission lines, have been extensively used [55,56]. It was found that an interdigital-capacitor-shaped defected ground structure-based microwave sensor has a higher sensitivity for the variations in relative permittivity, compared to other microwave sensors using a single-ring, a rotated single-ring, or a double-ring CSRR [56].

In this paper, gelatin’s humidity-sensing ability was compared with that of PVA at L-band (1~2 GHz) microwave frequencies. A high-sensitivity capacitive microwave sensor based on a defected ground structure with a modified interdigital capacitor (DGS-MIDC) in a microstrip transmission line resonating at 1.5 GHz without any coating was designed on an RF-301 substrate with a thickness of 0.76 mm [44]. Two microwave sensors coated with gelatin and PVA, respectively, with a thickness of 0.02 mm were fabricated. The first resonant frequency and the magnitude level of the transmission coefficient (S_21_) for the microwave sensors were used to compare the humidity-sensing characteristics when the RH was varied from 50% to 80% with a step of 10% at a temperature of 25 °C using a low-reflective temperature and humidity chamber manufactured with Styrofoam. All the simulated results in this paper were obtained using CST Studio Suite (Dassault Systèmes Co., Vélizy-Villacoublay, France) [57].

## 2. Microwave Sensor Geometry and Characteristic

**Figure 2:**
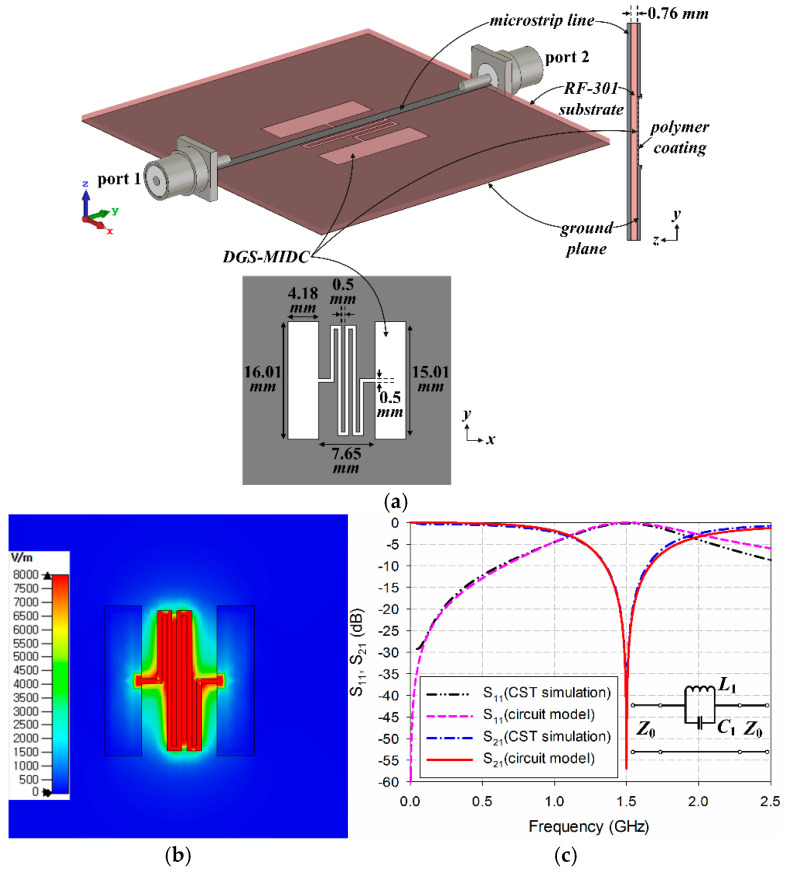
DGS-MIDC-based microwave sensor: (**a**) geometry, (**b**) electric-field distribution at 1.5 GHz, and (**c**) S-parameter characteristics and simplified equivalent circuit model.

A high-sensitivity two-port microwave sensor based on a DGS-MIDC was designed on a 50 mm × 50 mm RF-301 substrate with a relative permittivity (*ε*_r_) of 2.97, a loss tangent (tan *δ*) of 0.0009, and a thickness of 0.76 mm to concentrate the electric field along the microstrip transmission line, as shown in Figure 2a. The DGS-MIDC was etched on the ground plane of the microstrip transmission line with a width of 1.68 mm, and the geometric parameters were optimized to obtain the first resonant frequency of S_21_ at 1.5 GHz for unloaded conditions with a high sensitivity for relative permittivity. The proposed microwave sensor with the DGS-MIDC showed a band-stop characteristic at the resonant frequencies. When a capacitive polymer is coated on the top of the DGS-MIDC, the resonant frequencies of the microwave sensor change according to the variations in complex permittivity of the coated capacitive polymer for different RHs.

We note that the polymer-coated square area’s length was 18.01 mm, slightly longer than that of the DGS-MIDC, providing complete coverage, and the thickness of the coated area was measured to be 0.02 mm. The electric-field distributions were mainly concentrated along the slots between the interdigital fingers, as shown in Figure 2b. The simplified equivalent circuit model of the proposed IDCS-DGS considering only the first resonant frequency is shown as the inset in Figure 2c, and the circuit parameters can be obtained from the CST Studio Suite simulation results by using the following equations [58]:

(1) $$C_{1}=\frac{1}{Z_{0}}\frac{1}{4\pi \Delta f_{3\mathrm{dB}}}$$

(2) $$L_{1}=\frac{1}{{\left(2\pi f_{r}\right)}^{2}C_{1}}$$

where *Z*_0_ is the characteristic impedance of the transmission line, *f*_r_ is the resonant frequency, and ∆*f*_3dB_ is the 3 dB bandwidth at the first resonant frequency. The extracted circuit parameters for unloaded conditions are *C*_1_ = 1.6938 pF and *L*_1_ = 6.6464 nH, and the S-parameters obtained from these circuit parameters were compared with the simulation results in Figure 2c, which showed good agreement.

**Figure 3:**
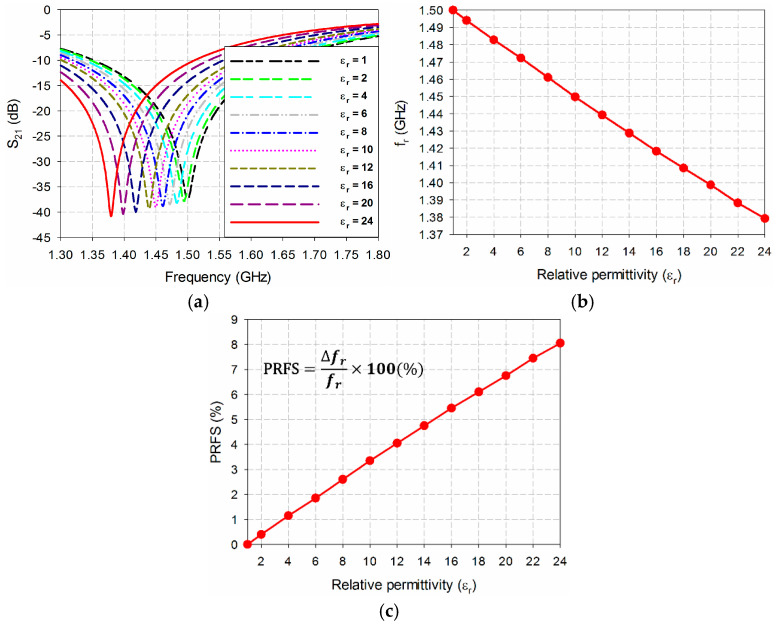
Performance characteristics of the DGS-MIDC-based microwave sensor for varying relative permittivity of the coated polymer with tan *δ* = 0: (**a**) S_21_, (**b**) *f*_r_, and (**c**) PRFS.

The changes in the first resonant frequency (*f*_r_) and the percent relative frequency shift (PRFS) of the S_21_ characteristics for the microwave sensor with the DGS-MIDC are shown in Figure 3. The PRFS is a percentage of the difference between the loaded and unloaded first resonant frequencies compared to the unloaded first resonant frequency. The coated polymer’s relative permittivity (*ε*_r_) changed from 1 to 24 for the lossless case (tan *δ* = 0) [44]. As the relative permittivity increased, the first resonant frequency decreased. For example, the first resonant frequency moved to 1.3793 GHz and its PRFS compared to the unloaded one at 1.5 GHz was 8.05% when the relative permittivity increased from 1 to 24.

Table 1 summarizes the simulated values of the first resonant frequencies and PRFSs for the different relative permittivities varying from 1 to 24.

It turned out that the variation in the PRFS in the first resonant frequency of S_21_ showed a nearly linear behavior for the varying relative permittivity from 1 to 24. Based on this knowledge, the relation of relative permittivity in terms of the PRFS was derived from a curve-fitting tool in Sigma Plot by using the following equation:

(3) $$\varepsilon_{r}=A_{0}+A_{1}\times PRFS+A_{2}\times {\left(PRFS\right)}^{2}$$

where $A_{0}=1.0075$, $A_{1}=2.5825$, and $A_{2}=0.0331$.

**Figure 4:**
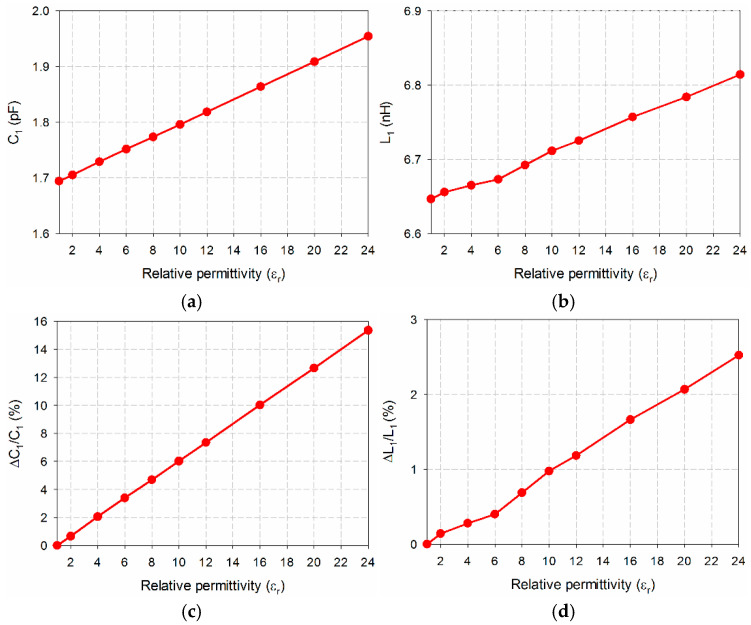
Extracted equivalent circuit parameters of the DGS-MIDC-based microwave sensor for varying relative permittivity of the coated polymer with tan *δ* = 0: (**a**) *C*_1_; (**b**) *L*_1_; (**c**) Δ*C*_1_/*C*_1_(%) and (**d**) Δ*L*_1_/*L*_1_(%).

Figure 4 shows the extracted equivalent capacitance and inductance, and their percent relative shifts when the relative permittivity of the MUT polymers varies from 1 to 24 with tan *δ* = 0. In this case, both the equivalent capacitance and inductance changed linearly. For instance, when the relative permittivity of the MUT was *ε*_r_ = 1, *C*_1_ and *L*_1_ of the proposed microwave sensor were 1.6938 pF and 6.6464 nH, respectively. As the relative permittivity of the MUT increased to *ε*_r_ = 24, *C*_1_ and *L*_1_ were increased to 1.9541 pF and 6.8141 nH, respectively. Therefore, Δ*C*_1_/*C*_1_(%) and Δ*L*_1_/*L*_1_(%) were 15.4% and 2.5%, respectively. Both *C*_1_ and *L*_1_ show nearly linear behaviors, but the variation in *C*_1_ is much higher compared to *L*_1_.

## 3. Experiment Results and Discussion

**Figure 5:**
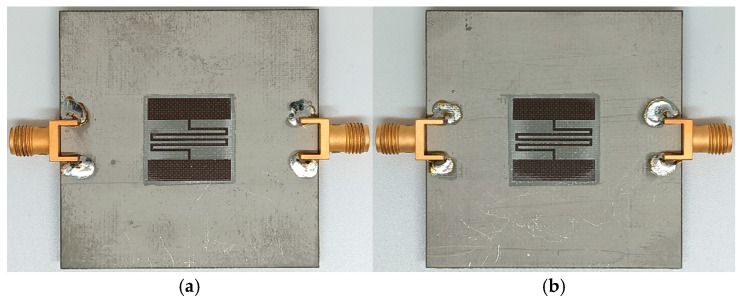
Photographs of the fabricated microwave sensors coated with (**a**) PVA and (**b**) gelatin.

Figure 5a,b shows photographs of the microwave sensors based on the DGS-MIDC coated with PVA and gelatin, respectively, fabricated on an RF-301 substrate. The PVA with a degree of polymerization of 1500 and a degree of saponification of 99 mol% was obtained from Yakuri Pure Chemicals Co., Ltd., Kyoto, Japan, whereas the type A gelatin (G1890-100G, gel strength ~300 g Bloom, from porcine skin) was provided by Sigma-Aldrich Co., St. Louis, MO, USA. The preparation of the polymer solutions with a concentration of 5 wt% was carried out by dissolving each polymer using deionized water as a solvent. First, each polymer solution of 55 mg was spread over the sensing area on the DGS-MIDC using a brush. Next, a convection oven (OF4-10P, JEIO TECH Co., Ltd., Daejeon, Republic of Korea) was used to dry the polymer-coated microwave sensors for 120 min at 60 °C. Finally, overnight drying in a vacuum oven (JSOV-30T, JS Research Inc., Gongju, Republic of Korea) was carried out at 80 °C. Field-emission scanning electron microscopy (S-4300, Hitachi High-Technologies Co., Ltd., Tokyo, Japan) was used at a voltage of 15 kV to measure the average thickness of the coated polymer film, and it was around 0.02 mm.

**Figure 6:**
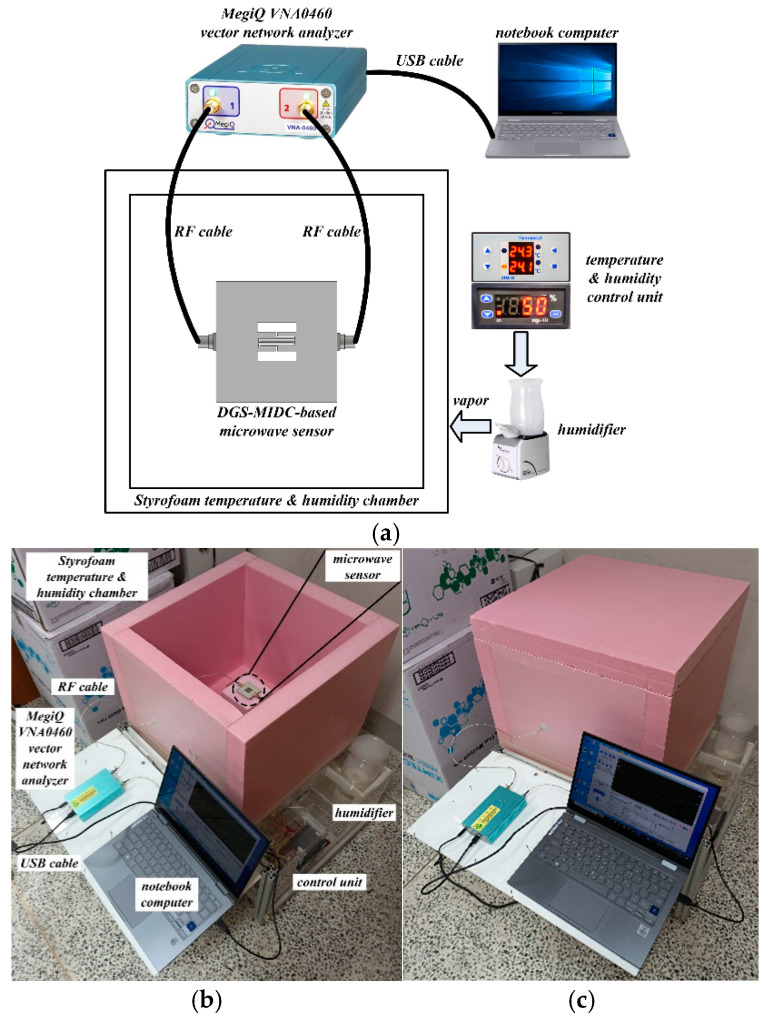
Block diagram and photographs of the experiment setup for the humidity-sensing measurements: (**a**) block diagram, (**b**) experiment setup with an open-top cover, and (**c**) experiment setup with closed-top cover.

For the humidity-sensing measurements, the experimental setup shown in Figure 6 was used. A custom-made low-reflective temperature and humidity chamber was manufactured using 50 mm thick Styrofoam walls, a thermostat for controlling temperature, a humidistat for controlling humidity, halogen lamps for adding temperature, and a humidifier for adding humidity. The MegiQ VNA-0460 (MegiQ, Eindhoven, The Netherlands) vector network analyzer was used to measure the S_21_ characteristics of the polymer-coated fabricated microwave sensors. The RH was increased from 50% to 80% with a step of 10% with a fixed temperature of around 25 °C. For the stabilization of the humidity inside the chamber along with ensuring water absorption into the polymer-coated area, the S_21_ characteristics were measured after waiting 10 min for each RH.

**Figure 7:**
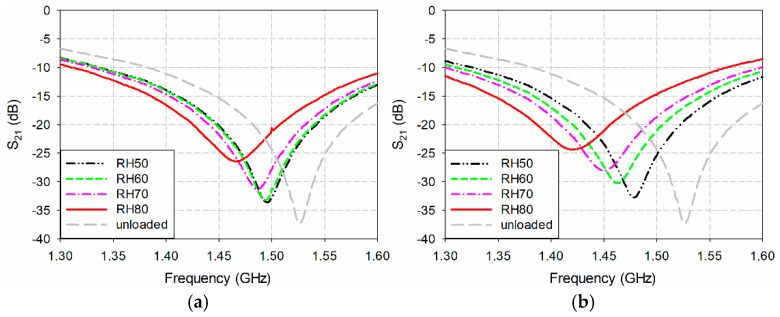
Measured S_21_ characteristics of the fabricated microwave sensors coated with the polymers for varying RH. (**a**) PVA and (**b**) gelatin.

The measured S_21_ characteristics of the fabricated microwave humidity sensors coated with PVA and gelatin, respectively, for each RH were compared in Figure 7. The measured first resonant frequency and magnitude level of the fabricated microwave sensors without a polymer coating (unloaded conditions) were 1.526 GHz and −37.38 dB, respectively. When the RH increased from 50% to 80% for the PVA-coated microwave sensor, the S_21_’s first resonant frequency moved from 1.496 GHz to 1.446 GHz, while its magnitude level increased from −33.69 dB to −27.79 dB. As the RH increased from 50% to 80% for the gelatin-coated microwave sensor, the S_21_’s first resonant frequency shifted from 1.478 GHz to 1.419 GHz, while its magnitude level increased from −32.82 dB to −24.41 dB. The values of the measured first resonant frequencies, PRFSs, magnitude levels, and percent relative magnitude shifts (PRMSs) of the fabricated microwave humidity sensors coated with PVA and gelatin are summarized in Table 2 and Table 3. Note that the PRMS is a percentage of the difference between the magnitude levels at the loaded and unloaded first resonant frequencies compared to the magnitude level at the unloaded first resonant frequency [44].

**Figure 8:**
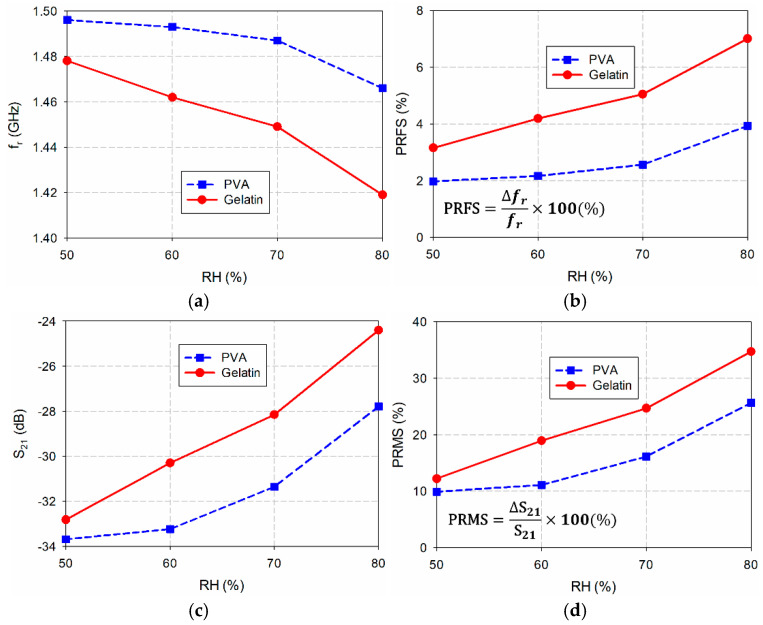
Performance comparison of the fabricated microwave sensors coated with the polymers for varying RH. (**a**) *f*_r_, (**b**) PRFS, (**c**) S_21_ magnitude, and (**d**) PRMS.

Figure 8 shows a plot of the measured first resonant frequencies, PRFSs, magnitude levels, and PRMSs of the fabricated microwave sensors coated with PVA and gelatin as a function of the RH, which are obtained from the results in Figure 7. The inset in Figure 8d shows the definition of PRMS. The PRFSs and PRMSs were calculated based on the first resonant frequency and magnitude level of the fabricated microwave sensor without any coating. When the RH increased from 50% to 80% for the PVA-coated microwave sensor, the PRFS increased 2.0 times from 1.97% to 3.93%, while the PRMS increased 2.59 times from 9.89% to 25.66%. As the RH increased from 50% to 80% for the gelatin-coated microwave sensor, the PRFS increased 2.23 times from 3.15% to 7.01%, while the PRMS increased 2.84 times from 12.21% to 34.71%. Accordingly, the capacitive humidity sensitivity of the gelatin-coated microwave sensor is higher compared to that of the PVA-coated one in terms of the PRFS and PRMS.

**Figure 9:**
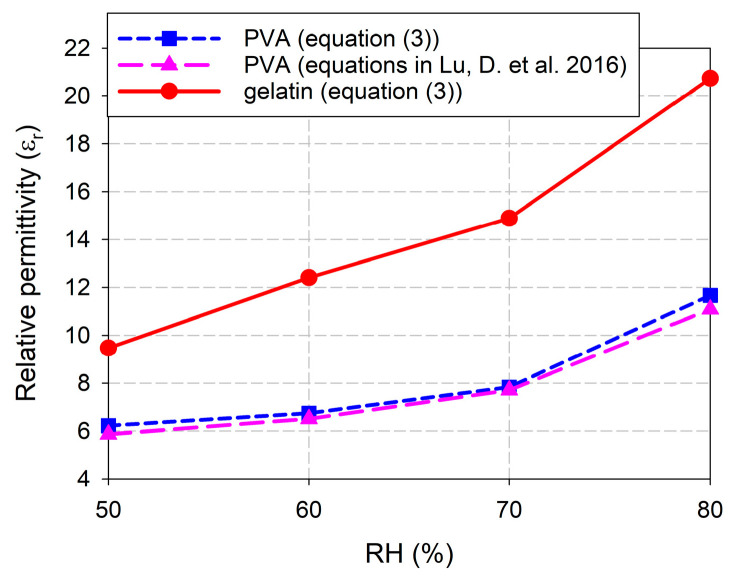
Comparison of the extracted relative permittivity from measured PRFSs of PVA- and gelatin-coated microwave sensors for varying RH.

Finally, the relative permittivity of the fabricated sensors coated with PVA and gelatin was extracted by using the measured PRFSs and Equation (3), as shown in Figure 9. When the RH increased from 50% to 80% for the PVA-coated microwave sensor, the relative permittivity increased 1.88 times from 6.22 to 11.67, while it increased 2.19 times from 9.47 to 20.74 for the gelatin-coated sensor. Note that the relative permittivity of PVA at around 1.5 GHz is around 4 to 5 [59], whereas that of gelatin is around 7 to 8 [60]. The relative permittivity of the PVA film for varying the RH can also be calculated by using equations in [61]. It increased 1.89 times from 5.87 to 11.09 when the RH increased from 50% to 80%, which shows a similar trend with slightly smaller values compared to that derived from equation (3). Therefore, we can conclude that the change in the extracted relative permittivity for the gelatin-coated microwave sensor is larger than that of the PVA-coated one for the varying RH.

## 4. Conclusions

We have compared the capacitive humidity-sensing performance of gelatin and PVA polymers using a planar microwave sensor based on a DGS-MIDC with a first band-stop frequency at 1.5 GHz without any coating. The variations in the first resonant frequency and the PRFS of the S_21_ characteristics were studied through a simulation when the coated polymer’s relative permittivity changed from 1 to 24 for the lossless case (tan *δ* = 0). The quadratic relationship between the coated polymer’s relative permittivity and the PRFS of the first resonant frequency was derived by using a curve fitting tool.

The prototypes of the microwave sensors coated with type A gelatin and PVA, respectively, with a thickness of 0.02 mm, were fabricated on an RF-301 substrate. When the RH was varied from 50% to 80% with a step of 10% at a temperature of 25 °C, the humidity-sensing characteristics of the gelatin- and PVA-coated microwave sensors were compared by using the changes in the resonant frequency and magnitude level of S21. For the PVA-coated microwave sensor, the PRFS increased 2.0 times from 1.97% to 3.93% when the RH increased from 50% to 80%, whereas it increased 2.23 times from 3.15% to 7.01% for the gelatin-coated microwave sensor. The PRMS increased 2.59 times from 9.89% to 25.66% as the RH increased from 50% to 80% for the PVA-coated microwave sensor, whereas it increased 2.84 times from 12.21% to 34.71% for the gelatin-coated microwave sensor. Therefore, the sensitivities of the gelatin-coated microwave sensor in terms of the PRFS and PRMS are higher compared to those of the PVA-coated microwave sensor.

A similar trend was observed in the variations of the extracted relative permittivity based on the measured PRFSs. For the PVA-coated microwave sensor, the extracted relative permittivity increased 1.88 times from 6.22 to 11.67 when the RH increased from 50% to 80%, whereas it increased 2.19 times from 9.47 to 20.74 with a larger change for the gelatin-coated sensor.

Therefore, gelatin can be a potential candidate for a high-sensitivity natural capacitive humidity-sensing material to replace PVA for various microwave sensors, such as chipless RFID tags, wireless sensors, or wearable sensors. The mechanical, physical, and chemical stability, repeatability, and reliability of gelatin and PVA need to be explored further in future work. We also plan to compare the humidity-sensing performances between type A and type B gelatins.

## Figures and Tables

**Table 1:** First resonant frequencies and PRFS of S_21_ responses of the DGS-MIDC-based microwave sensor for varying relative permittivity of the coated polymer with tan *δ* = 0.

	*ε*_r_ = 1	*ε*_r_ = 2	*ε*_r_ = 4	*ε*_r_ = 6	*ε*_r_ = 8	*ε*_r_ = 10	*ε*_r_ = 12	*ε*_r_ = 14	*ε*_r_ = 16	*ε*_r_ = 18	*ε*_r_ = 20	*ε*_r_ = 22	*ε*_r_ = 24
*f*_r_ (GHz)	1.5	1.4940	1.4828	1.4723	1.4610	1.4497	1.4393	1.4288	1.4183	1.4085	1.3987	1.3883	1.3793
PRFS (%)	0	0.40	1.15	1.85	2.60	3.35	4.05	4.75	5.45	6.10	6.75	7.45	8.05

**Table 2:** Measured first resonant frequencies, PRFSs, magnitude levels, and PRMSs of S_21_ responses of the DGS-MIDC-based microwave sensor coated with PVA for varying RH.

PVA	Unloaded	RH50	RH60	RH70	RH80
*f*_r_ (GHz)	1.526	1.496	1.493	1.487	1.466
PRFS (%)	0	1.97	2.16	2.56	3.93
S_21_ (dB)	−37.38	−33.69	−33.24	−31.36	−27.79
PRMS (%)	0	9.89	11.09	16.12	25.66

**Table 3:** Measured first resonant frequencies, PRFSs, magnitude levels, and PRMSs of S_21_ responses of the DGS-MIDC-based microwave sensor coated with gelatin for varying RH.

**Gelatin**	**Unloaded**	**RH50**	**RH60**	**RH70**	**RH80**
*f*_r_ (GHz)	1.526	1.478	1.462	1.449	1.419
PRFS (%)	0	3.15	4.19	5.05	7.01
S_21_ (dB)	−37.38	−32.82	−30.30	−28.15	−24.41
PRMS (%)	0	12.21	18.95	24.69	34.71

## Data Availability

Data are contained within the article.
